# Listening first: mapping the inner game of an elite women's rugby national team and its staff

**DOI:** 10.3389/fpsyg.2026.1927634

**Published:** 2026-09-23

**Authors:** Alessia Pecchini, Claudio Robazza, Francesca Vitali

**Affiliations:** 1 Department of Neurosciences, Biomedicine and Movement Sciences, University of Verona, Verona, Italy; 2 Department of Medicine and Aging Sciences, BIND-Behavioral Imaging and Neural Dynamics Center, “G. d'Annunzio” University of Chieti-Pescara, Chieti, Italy

**Keywords:** applied sport psychology, elite women's rugby, initial evaluation, psychological needs assessment, women athletes

## Abstract

**Introduction:**

A well-developed intake assessment can identify athletes' and staff members' initial psychological needs and thus lay the foundation for targeted, evidence-based sport psychology interventions. However, empirical descriptions of such needs in elite women's collision-sport settings remain scarce.

**Methods:**

In collaboration with the Italian Rugby Federation, the Italian Women's Rugby National Team, and its staff, this study used a mixed-methods intake assessment protocol to identify the priority psychological needs of the team. Qualitative results from 32 athletes and 12 staff members showed cohesion as the primary team need requiring strengthening. Individually, players most frequently requested improvements in self-confidence and emotional regulation.

**Results:**

Quantitative findings revealed low self-rated mental health scores among both athletes and women staff members, underscoring the importance of mental health support. Among staff, gender was associated with higher stress (women reported greater stress), and having a dual career, defined as the successful combination of education, training, and/or work with sport, was associated with higher happiness.

**Discussion:**

Taken together, these results offer sport psychologists a clear, participant-driven map of the psychological targets to address in elite women's team sports, directly informing the design and evaluation of future tailored interventions.

## 1 Introduction

A sport psychologist should be fully integrated into the high-performance environment, operating as a member of the staff with equal recognition alongside technical, physical conditioning, video-analysis, medical, managerial, and social-media-communication personnel (Reid et al., 2004). Indeed, to understand elite athletes' and coaches' personalities, attitudes, interpersonal and intrapersonal skills, and relationships during training and competition, a sport psychologist should observe and evaluate their behaviors in a natural environment. This allows for the development of support interventions tailored to specific psychological needs (Andersen, 2000).

In the context of applied sport psychology, psychological needs refer to the specific cognitive, emotional, and social resources, such as psychological skills, wellbeing support, and coping mechanisms, required by athletes and staff members to bridge the gap between their current state and their optimal functioning (Taylor, 2017). Identifying these baseline needs is a critical prerequisite for effective intervention planning. To capture a comprehensive profile of the team, the design of our initial needs assessment was guided by the Holistic Athletic Career Model (Wylleman, 2019). The Holistic Athletic Career Model emphasizes that athletes face concurrent demands and normative transitions across athletic, psychological, psychosocial, and academic/vocational domains. A key element of the vocational domain is the concept of a dual career, defined as the successful combination of education, training, or work with high-performance sport (Stambulova and Wylleman, 2014). Exploring these dual-career pathways is crucial, as successfully navigating them acts as a protective factor for wellbeing and life satisfaction. Furthermore, the psychosocial layer of this model highlights the essential role of the athletes' interpersonal network, including coaches and support staff. Consequently, an effective needs assessment must encompass these key figures and explore not only sport-specific objectives but also life and vocational goals, ensuring a truly holistic evaluation.

Simultaneously, defining what these needs aim to achieve requires a structured understanding of goals. Goal-setting is defined as the process of establishing specific, measurable, and time-bound targets that direct attention, mobilize effort, and motivate the development of new strategies (Locke and Latham, 2002). In applied settings, collaboratively defined goals derived directly from a participant-driven needs assessment, rather than imposed externally, foster higher commitment and strengthen the working alliance. Thus, by combining the holistic career perspective (to understand the multidimensional demands and the psychosocial network) with goal-setting (to define the actionable targets of the intervention), practitioners can design highly tailored support strategies.

Several authors emphasize the importance of starting with a thorough understanding of a client's initial psychological needs, thoughts, and behaviors to ensure intervention effectiveness and to actively involve the participant in the process (Anderson et al., 2002; Moore, 2003). This approach allows practitioners to collect information while building a relationship with the client (Rosqvist et al., 2007). The working alliance has been shown to account in therapy for 30% of the variance in positive outcomes (Asay and Lambert, 1999), making it a key predictor of success. Despite this emphasis, practical examples and comprehensive evidence-based procedures for conducting initial needs evaluations in sport psychology counseling remain scarce (Sharp and Hodge, 2011).

Early attempts to address this gap include the *Sport-Clinical Intake Protocol* (SCIP) developed by Taylor and Schneider (1992). However, the SCIP was criticized for being too clinically oriented for athletes seeking performance-enhancement interventions (Simons, 2010). Consequently, in 2017, the *Performance Interview Guide* (PInG) was developed to help sport psychologists conduct initial consultations and create an effective working relationship for sport performance enhancement interventions (Aoyagi et al., 2017). In the same year, Taylor (2017) proposed an updated version of the SCIP, named *Sport Interviewing Protocol* (SIP), specifically designed to collect a wide range of information to comprehensively understand clients' needs in the sport-specific context and, consequently, to develop an effective sport psychology applied intervention.

Comprehensive protocols for initial needs assessment of clients offer several advantages. They can be administered and scored consistently across groups of athletes, enabling the development of comparative norms both between and within athletes. Unlike standardized tests, the qualitative and open-ended nature of the questions allows for a more detailed and nuanced exploration of athletes' and staff members' psychological experiences, including their behaviors, thoughts, and emotions, and enables the practitioner to follow up on significant clues and insights that arise during the assessment. Qualitative assessment can also build rapport and trust between the consultant and the client.

Despite these advantages, qualitative assessment also presents some disadvantages, such as being inherently subjective in the collection and interpretation of the gathered information, making it vulnerable to possible cognitive biases. To mitigate this issue, incorporating closed-ended questions, such as those using Likert-type scales, can provide quantitative data in addition to qualitative information, offering a more balanced and comprehensive understanding of the client's needs (Taylor, 2017).

### 1.1 Elite women's rugby specificity

Women's participation in sports has grown worldwide over the last decades (Eime et al., 2020). This positive trend also holds for sports typically associated with men, such as rugby and football, two of the most popular team sports worldwide. Indeed, World Rugby considers women's participation as the greatest opportunity to attract new players and fans and to expand rugby's participation: according to World Rugby data, women's rugby has grown rapidly globally, and the women's game has greater value, visibility and viability (World Rugby, 2026).

One of the main challenges for sport psychologists working in elite women's rugby is the lack of an established tradition for the profession within this sport, together with a more negative perception of sport psychologists in physical-contact, collision-based, and combat sports compared to non-contact sports (Green et al., 2012). Fears of being negatively stereotyped for disclosing psychological distress or mental health symptoms may prevent elite athletes from seeking help. Moreover, being an elite woman athlete can itself represent a risk factor for poor mental health. Indeed, Küttel and Larsen (2020) published a scoping review on risk and protective factors for mental health in elite athletes and reported that elite women athletes reported a higher risk of common mental health disorders than elite men athletes, as other scholars have also highlighted (Appaneal et al., 2009; Schaal et al., 2011).

Regardless of gender, elite athletes may worry about being perceived as weak-minded, which could damage their sporting reputation (Reardon et al., 2019). However, for elite women's rugby players, the requirement for robust psychological skills, particularly emotional regulation, confidence, and stress management, is amplified by the unique demands of performing in a high-impact, collision sport while simultaneously navigating the systemic and sociocultural challenges historically associated with women's sports. Specifically, women competing in traditionally male-dominated collision sports like rugby often face intense gendered societal expectations. This can lead to “apologetic” behaviors as athletes attempt to reconcile the physical aggressiveness of the sport with traditional norms of femininity (Hardy, 2014). Furthermore, these athletes frequently contend with structural inequities, such as sub-optimal coaching environments and resources that represent just the “bare minimum” compared to the men's game (Dane et al., 2023). Understanding and targeting these specific psychological needs is therefore not only a performance enhancer but a vital protective factor for their overall wellbeing. Accordingly, the contribution of sport psychologists in identifying and supporting elite athletes presenting mental health concerns is increasingly recognized. As (Reardon et al., 2019) highlighted in the International Olympic Committee's position statement on elite athlete mental health, evidence-based sport psychology counseling interventions are crucial. When sport-specific interventions have been conducted, they have successfully improved the recognition of mental health symptoms and disorders, increased professional referral knowledge, reduced stigma, and enhanced help-seeking intentions (Breslin et al., 2017). This growing recognition is reflected in numerous recent position statements by sport psychology societies and associations on mental-health protection and promotion in sport (see Gorczynski et al., 2019; Moesch et al., 2018; Schinke et al., 2018; Van Slingerland et al., 2019; Vella et al., 2021).

Despite the recognized importance of the working alliance and the theoretical consensus on holistic athlete development, a critical research gap remains: the applied sport psychology literature currently lacks empirical, methodological blueprints demonstrating how to systematically evaluate and map these initial baseline needs within a holistic framework.

### 1.2 Study purpose

To address the gap in the literature between theoretical models of elite women athletes and staff members wellbeing and the practical design of applied interventions, this study providing a transparent, participant-driven example of an intake evaluation aimed to identify the initial psychological needs of elite women's rugby players and their staff members as a direct foundation for a future targeted, evidence-based, and tailored sport psychology intervention. A mixed-methods intake assessment protocol was specifically chosen for its ability to provide a comprehensive profile; qualitative data captured the nuanced, participant-driven narratives of their experiences, while quantitative scales provided structured, self-report indicators regarding baseline mental health and wellbeing. Through semi-structured interviews with the Italian Women's Rugby National Team and its staff, the primary applied objective was to prioritize the specific goals emerging directly from the participants' perspectives, which will serve as focal aims for subsequent sport psychology counseling.

In particular, this study addressed these questions: (1) What previous experiences and expectations regarding sport psychology did athletes and staff members report? (2) What personal, sport-related, team, and dual-career goals did participants identify? (3) What individual- and team-level psychological needs emerged from the interviews, and where did athletes' and staff members' perspectives converge or diverge? (4) How did participants rate physical health, mental health, happiness, stress, coping, and social support, and what exploratory associations or subgroup differences were observed?

Beyond informing a specific intervention, this research makes a significant scientific contribution to the literature by offering a replicable example for conducting system-level needs evaluations, providing practitioners and researchers with an empirical framework to assess psychological targets in elite team sports.

## 2 Method

### 2.1 Eligibility criteria

This study adhered to the ethical guidelines set forth in the Declaration of Helsinki and gained approval from the Ethics Committee of the University of Verona (nr. #45.R1/2024). The eligible athletes pool consisted of all 32 players representing the Italian Women's rugby union national team participating in the 2025 edition of the Women's Six Nations Championship (comprising the 25 officially selected roster players alongside 7 invited players). Additionally, core staff members (i.e., technical, physical conditioning, video-analysis, medical doctors, managerial, and social-media-communication staff members) were eligible to participate. It should be noted that the team physiotherapists were deliberately excluded from the data collection due to high personnel turnover during that specific period. Because they had not been integrated into the environment long enough to accurately observe and assess the team's established psychosocial dynamics, they were deemed unable to provide adequately informed responses.

Because this study functioned as an exploratory, whole-team needs assessment rather than a hypothesis-testing experiment, the maximum possible sample size was inherently predetermined by the team's official roster. Given this purposive, population-level sampling of a specific intact elite ecosystem, an *a priori* power analysis to determine sample size was neither applicable nor necessary for the study's aims (Eldridge et al., 2016).

### 2.2 Participants

The total sample comprised 32 athletes, ranging in age from 18 to 33 years (M_age_ = 26.4; SD = 4.1 years), and 12 staff members (4 women and 8 men; ranging in age from 30 to 56 years; M_age_ = 41.3; SD = 8.1 years). The majority of the athletes (30 out of the 32) reported having a dual-career path. Dual career is intended as a successful combination of education/academic study, training, and/or work with sport (Wylleman et al., 1999).

The staff members included four coaches (one woman and three men), two physical conditioning trainers (men), one video analyst (man), two physicians (women), two managers (one woman team manager and one man rugby director), and one social media communication expert (man). Four staff members out of the twelve reported having a dual-career path (three women and one man).

Players were asked to report their rugby experience since the start of their career (M_exp_rugby_ = 13.9; SD = 4.8 years), and the first participation in the Italian Women's Rugby National Team (M_exp_national_ = 6.5; SD = 3.8 years). Staff members' experience in their individual roles was also reported (M_exp_roles_ = 13.5; SD = 7.7 years), as well as their participation as staff members of the Italian Women's Rugby National Team (M_exp_national_ = 6.1; SD = 5.1 years).

### 2.3 Procedure and study design

This study employed a convergent mixed-methods design to simultaneously capture in-depth narratives and objective baseline metrics. We conducted 44 (32 athletes and 12 staff members) online interviews via online Zoom platform (Zoom Video Communications Inc., 2024) during the pre-competition phase, at times convenient for the participants' schedules. Prior to each interview, participants were informed about the objectives of the study and provided verbal consent to participate and to record the interview. Anonymity and voluntary participation were assured. The interviews were conducted together by the first and the last author, both of whom are licensed sport psychologists. The last author has extensive applied experience in elite sports, while the first author brings contextual understanding and rapport-building capabilities as a former elite athlete. To ensure methodological consistency and rigor across all 44 sessions, the researchers conducted the interviews jointly and maintained a standardized division of roles: the last author initiated each session, and the first author continued and closed it.

The semi-structured interview guide consisted of two different revised versions of the *Sport Interviewing Protocol* (SIP; Taylor, 2017): one for athletes and one for staff members (see Supplementary Material). Following Taylor's (2017) recommendation to tailor the SIP to the specific research/intervention approach and the needs and goals of the individuals involved, we retained the core open-ended questions addressing previous experiences with sport psychology, personal and athletic goals, and initial needs assessment (e.g., “What are your personal athletic/life goals this season?”, “Which aims do you expect psychological counseling will be focused on?”, and “How would you describe your relationship with your national teammates?”), and modified the protocols by adding closed-ended questions (rated on a 10-point Likert-scale, ranging from 1 = very, very low to 10 = very, very high). These closed-ended questions quantitatively evaluated areas such as physical health, mental health, and social support (e.g., “How would you rate your general physical health?”, “How would you rate your general happiness?”, and “How satisfied are you with the support you receive from your family within sport?”).

Athletes' interviews (M_duration_ = 57.53; SD = 14.59 min) were longer, on average, than staff members' interviews (M_duration_ = 45.04; SD = 18.07 min), as the athletes' interview protocol included a greater number of questions (e.g., support from the staff members; support from the team).

### 2.4 Data analysis

#### 2.4.1 Qualitative analysis

Interviews were audio-recorded and transcribed verbatim. To address Research Questions 1, 2, and 3, qualitative data were processed through an inductive Descriptive Content Analysis approach (Elo and Kyngäs, 2008), ensuring that categories and key psychological themes emerged directly from the athletes' and staff members' narratives rather than from pre-defined theories. The process involved three phases: (1) preparation (i.e., immersion in the data), (2) organization (i.e., open coding, creation of categories, and abstraction), and (3) reporting. Data were managed using Delve software (Ho and Limpaecher, 2020), to systematically map the players' needs.

To ensure methodological transparency and rigor, the first author led the preparation and initial organization phases, engaging in repeated active reading to become intimately familiar with the transcripts before assigning open codes. To establish reliability, the study utilized investigator triangulation (Patton, 2015). Throughout the analytical process, the initial codes, emerging categories, and final abstractions were continuously reviewed and debated during regular research team meetings. The co-authors critically evaluated the first author's initial categorizations, and any disagreements in data interpretation were resolved through collaborative discussion until a mutual consensus was reached. Following this inductive process, categories were aggregated into overarching themes, and their frequency distribution was calculated to highlight the most prevalent psychological needs. This methodology allowed for a rigorous quantification of recurring concepts, providing an objective representation of the team's psychological requirements (Moretti et al., 2011). As participants were free to provide multiple responses or to withhold opinions on specific topics, the number of responses (n) varies across sections and does not always equal the total sample size; this flexibility is inherent in our participant-centered approach.

#### 2.4.2 Quantitative analysis

To complement the qualitative insights and address Research Question 4 (evaluating baseline psychosocial and physical health profiles, exploratory associations, and subgroup differences), qualitative data were integrated with quantitative metrics obtained from closed-ended questions. Prior to running inferential statistics, the assumptions of the tests, including normality (Shapiro-Wilk test) and homogeneity of variances (Levene's test), were systematically checked.

As an exploratory analysis to identify potential contextual trends, independent samples *t*-tests were conducted. These tests compared self-rated mental health variables (e.g., happiness, stress) and physical health metrics (e.g., sleep, eating behaviors) across specific sub-groups, namely between women and men staff members, and between staff members with and without a dual career. Furthermore, Pearson's bivariate correlations were performed across all quantitative variables, separately for the two samples (players and staff), to broadly explore the interplay between continuous psychological and physical health metrics. All quantitative data were analyzed via JASP software, version 0.19.1 (The JASP and Team, 2024), and Cohen's (2013) conventions were employed to interpret the effect sizes.

## 3 Results

The findings are divided into five main categories, following the sections of the intake protocol: (1) previous experiences in sport psychology and expectations, (2) personal and sport-related goal setting, (3) identification of the psychological needs, (4) perceptions of support, and (5) physical and mental health evaluations.

For each category, the aim was to highlight the specific elements that athletes and staff members identified as an expectation or a necessity, both for the individual and for the group.

### 3.1 Previous experience in sport psychology and expectations

Understanding participants' previous experiences and preconceived notions is crucial, as these shape the working alliance (Taylor, 2017). Overall, the introduction of a sport psychology intervention was met with a proactive and engaged attitude across the board.

#### 3.1.1 Expectations of support

Despite a portion of the athletes having no prior experience with sport psychology, resistance was minimal. The majority of athletes viewed the intervention as a vital resource for personal and emotional support, noting that the demands of their competitive level meant they “can no longer manage it alone” (Table 1). Athletes specifically expected the intervention to provide a safety net for mental downturns and to serve as a unifying force for the team. Only a small subset explicitly expected interventions focused purely on performance enhancement.

**Table 1:** Summary of the qualitative analysis of athletes and staff members' initial needs.

Category	Sub-category	Group	Frequency
Expectations	Seeking personal/emotional support	Athletes	37.5%
	Need for role clarity & integration	Staff	58.3%
Goal setting	Lack of shared, clear team goals	Athletes Staff	37.5% (A) 58.3% (S)
	Navigating dual-career demands	Athletes	93.75% engaged in dual-career
Psych. needs (Indiv.)	Low self-confidence/self-esteem	Athletes	53.1%
	High levels of general anxiety	Athletes	43.7%
	Difficulty with mistake management	Athletes	40,6%
	Respect for roles & professionalism	Staff	66.7%
Psych. needs (Team)	Severe lack of group cohesion	Athletes Staff	81.2% (A) 50.0% (S)

#### 3.1.2 Staff perspectives and risks

The performance staff members, drawing on their prior experiences with psychological interventions, approached the collaboration as an opportunity to build culture. However, their primary concern centered on role clarity (Table 1). Staff members emphasized the importance of athletes understanding that the sport psychologist is not a clinical “personal psychologist” for off-field problems, but a recognized member of the performance staff. Staff also acknowledged the inherent vulnerabilities of the athletes, noting that the growing demands of the sport have generated stress that players must learn to manage.

### 3.2 Personal and sport-related goal setting

To contextualize the athletes' current challenges, personal and sport-related goals were explored. These goals revealed the heavy external demands placed on the players, as well as a critical gap in collective planning.

#### 3.2.1 Athletes performance and dual-career demands

Individual athletic goals were heavily skewed toward physical rehabilitation, improving fitness, and securing a starting position (Table 1). However, a notable portion of the athletes admitted they struggle to set and pursue concrete personal goals. Furthermore, these athletic ambitions were pursued alongside immense external pressures; the vast majority of the team was navigating a dual career. Consequently, a primary goal for many athletes was simply managing the tiring logistics of balancing work and training, while others were actively bracing for impending career transitions.

#### 3.2.2 Systemic and team goals

A critical finding emerged regarding collective objectives. While athletes inherently focused on result-oriented milestones (e.g., qualifying for the Women's World Cup), there was a strong consensus across both athletes and staff members regarding the absence of shared, structured team goals (Table 1). Athletes noted that goals were “not clear and shared,” a sentiment strongly echoed by the staff members, who stressed the immediate need for a unified work plan to guide the team regardless of external dynamics.

### 3.3 Identification of psychological needs

Equipped with the contextual data regarding goals and expectations, specific targets for psychological intervention were identified, categorized into individual and team-level needs.

#### 3.3.1 Individual psychological needs

The assessment highlighted a pronounced need for cognitive and emotional regulation among the athletes. Over half of the players identified a need to rebuild self-confidence and self-esteem, often expressing feelings of inadequacy regarding their roster spots (Table 1). Furthermore, athletes reported frequent struggles with managing general anxiety and handling in-game mistakes. Rather than vocalizing these struggles, athletes typically internalize them. Conversely, the staff members identified different individual priorities for the players, focusing primarily on professionalism, respecting roles, and mitigating the athletes' tendency to withdraw and isolate themselves during conflicts.

#### 3.3.2 Team psychological needs

At the collective level, interpersonal dynamics were the primary concern. A severe lack of group cohesion was overwhelmingly identified as the team's greatest psychological need by both athletes and staff members (Table 1). Participants described a fractured environment “divided into small groups.” For the athletes, repairing this fracture meant restoring a sense of enjoyment and fun on the pitch, alongside improving on-field communication and addressing the lack of competence in current leadership roles. The staff members mirrored this need for a cultural shift, though they framed it around building a more rigorous professional attitude and commitment toward the national team.

### 3.4 Perceptions of support

Athletes and staff members operate within a broad social system that significantly impacts wellbeing and performance. Identifying the quality of these relationships helps contextualize performance challenges.

#### 3.4.1 Athletes

Players reported the highest perceived support from family (*M* = 8.89 ± 2.02) and friends (*M* = 8.02 ± 1.55). Within the organizational structure, support scores were highest for management (*M* = 8.78 ± 2.60) and performance staff members (*M* = 8.13 ± 1.85), whereas lower support ratings were reported for coaches (*M* = 6.36 ± 1.20) and medical staff (*M* = 6.10 ± 1.79); (see Table 2).

**Table 2:** Descriptive statistics and Pearson's correlations of athletes' and staff members' individual perceptions on considered variables.

Variable	Participant	M±SD	1		2		3		4		5		6		7		8
1. General health	A	7.34 ± 1.4	–														
	S	7.54 ± 1.78	–														
2. Training	A	7.58 ± 1.22	0.660	^***^	–												
	S	5.25 ± 2.93	0.146														
3. Sleep	A	6.56 ± 2.1	0.440	^*^	0.430	^*^	–										
	S	6.37 ± 1.68	−0.333		0.183		–										
4. Eating behavior	A	7.06 ± 1.55	0.600	^***^	0.548	^**^	0.202		–								
	S	6.17 ± 2.24	−0.276		0.243		0.145		–								
5. Social support	A	8.43 ± 1.74	0.007		0.164		−0.098		0.165		–						
	S	8.29 ± 2.16	0.275		−0.005		0.606	^*^	−0.378		–						
6. Mental health	A	6.84 ± 1.03	0.482	^**^	0.306		0.495	^**^	0.559	^***^	−0.020		–				
	S	6.29 ± 1.78	−0.050		−0.142		0.468		−0.063		0.581	^*^	–				
7. Happiness	A	7.28 ± 1.42	0.334		0.293		0.474	^**^	0.372	^*^	0.008		0.758	^***^	–		
	S	7.5 ± 1.09	0.435		−0.043		0.162		−0.093		0.668	^*^	0.622	^*^	–		
8. Stress^a^	A	6.04 ± 1.94	−0.013		−0.134		0.062		−0.282		−0.376	^*^	−0.119		−0.143		–
	S	6.94 ± 1.54	−0.312		−0.117		0.467		−0.023		0.320		0.884	^***^	0.188		–
9. Coping	A	7.05 ± 1.3	−0.151		−0.165		0.099		−0.269		−0.304		0.030		−0.090		0.057
	S	7.25 ± 1.83	0.724	^**^	−0.072		−0.211		−0.133		0.366		0.227		0.686	^*^	−0.122

M, mean; SD, standard deviation; A, athletes; S, staff members.

^*^p < 0.05.

^**^p < 0.01.

^***^p < 0.001.

^a^reversed value.

#### 3.4.2 Staff

Staff members reported high mean satisfaction regarding support from their personal social systems (*M* = 8.23 ± 2.16) (Table 2). Independent samples *t*-tests indicated no significant differences in perceived support based on gender (*t* = 0.597, *p* = 0.28) or dual-career status (*t* = −0.644, *p* = 0.27).

### 3.5 Physical and mental health evaluations

#### 3.5.1 Athletes

Individual physical health ratings indicated highest scores for training level (*M* = 7.58 ± 1.22) and general health (*M* = 7.34 ± 1.40). Individual mental health scored (*M* = 6.84 ± 1.03), and individual stress scored (*M* = 6.04 ±1.94; see Table 2). Team-level evaluations yielded lower scores for team mental health (*M* = 5.62 ± 1.51) and team happiness (*M* = 5.62 ± 1.44), while team stress (*M* = 7.31 ± 1.10) and team coping strategies scored (*M* = 6.06 ± 1.70). Bivariate correlation analyses indicated that team mental health correlated positively with team happiness (*p* < 0.001) and collective coping strategies (*p* < 0.05) (see Table 3).

**Table 3:** Descriptive statistics and Pearson's correlation of athletes' team perceptions on considered variables.

Variable	M±SD	1		2		3		4		5		6		7
1. Coach support	7.34 ± 1.4	–												
2. Performance coaches support	7.58 ± 1.22	0.290		–										
3. Medical staff support	6.56 ± 2.1	0.330		0.291	^*^	–								
4. Manager support	7.06 ± 1.55	0.280		0.232	^**^	0.230		–						
5. Team mental health	5.62 ± 1.51	0.430	^*^	0.373	^*^	0.322		0.188		–				
6. Team happiness	5.62 ± 1.44	0.139		0.487	^**^	0.438	^*^	0.012		0.640	^***^	–		
7. Team stress^a^	7.31 ± 1.1	0.229		−0.086		0.191		−0.166		0.112		0.061		–
8. Team coping	6.06 ± 1.7	0.158		0.404	^*^	0.180		−0.059		0.446	^*^	0.702	^***^	−0.157

M, mean; SD, standard deviation; A, athletes; S, staff members.

^*^p < 0.05.

^**^p < 0.01.

^***^p < 0.001.

^a^reversed value.

#### 3.5.2 Staff

Staff members' general health averaged (*M* = 7.54 ± 1.78) and individual happiness averaged (*M* = 7.50 ± 1.09). Significant demographic variations were observed: a dual-career effect emerged for training level (*t* = 2.181, *p* < 0.05), with dual-career staff reporting lower scores than single-career staff, and for happiness (*t* = −1.873, *p* < 0.05), where dual-career staff reported higher scores (Table 2). Furthermore, a significant gender effect was found for individual stress (*t* = −3.094, *p* < 0.01), with women staff members reporting higher stress levels than men staff members.

## 4 Discussion

This study presented the qualitative and quantitative results of a comprehensive intake assessment protocol aimed at identifying the specific baseline needs of elite women rugby players and their staff. While the broader literature extensively documents various sport psychology interventions, there remains a distinct scarcity of published examples detailing how to systematically conduct and approach a comprehensive needs assessment at the team level. This study addresses this gap by utilizing a multi-stakeholder, mixed-methods intake protocol, simultaneously capturing the perspectives of both athletes and multidisciplinary staff members in a high-impact collision sport (i.e., women's rugby). By bridging qualitative thematic needs with quantitative wellbeing metrics, this research offered a practical framework that moves beyond generalized psychological profiling. The findings concurred with broader literature while offering new perspectives on the unique structural, emotional, and cultural demands of women's national teams.

To address the Research Question (RQ) 1, which explored participants' previous experiences and expectations regarding sport psychology, our findings highlighted the importance of the initial approach. As Taylor (2017) suggested, beginning with targeted questions on expectations and concerns helps foster positive attitudes and an open mind. This was observed in both the players and staff members, who expressed genuine interest in this collaboration.

In addressing RQ 2, focusing on the personal, sport-related, team, and dual-career goals identified by participants, cohesion emerged as a primary collective target from both the players' and staff members' perspectives. Being part of a national team means having less time together than in a club setting, making strong team cohesion more difficult to build. In line with Goal-Setting Theory (Locke and Latham, 2002), defining such targets collaboratively helps direct attention and mobilize effort. Indeed, (Tod et al., 2024) suggest that an athlete-centered procedure empowers athletes to actively participate in developing their team's goals, positively influencing team cohesion. Furthermore, concerning dual-career goals, the heavy demands and impending transitions reported by the athletes (with 75% of women in the sample pursuing dual career paths) align with the vocational domain of the Holistic Athletic Career Model (Wylleman, 2019), highlighting that educational and professional goals must be integrated into the psychological profiling of elite athletes.

To answer RQ 3, which examined individual- and team-level psychological needs and the convergence of perspectives, thematic analysis showed that athletes most frequently requested individual work on self-confidence and self-esteem. This aligns with previous findings indicating that women athletes tend to report lower levels of sport-specific self-confidence than men athletes, and that self-confidence is a key factor influencing athletic performance (Clifton and Gill, 1994). Relatedly, emotion regulation, especially after mistakes, emerged as another prominent individual need. Extant research suggests that women athletes often experience negative emotions accompanied by harsh self-scrutiny and self-criticism. Moreover, they may face additional challenges related to self-criticism, such as fear of failure, perfectionism, body-related concerns, and anxiety (Killham et al., 2018). In a study of ego- and task-orientation in 196 women volleyball players, Voight et al. (2000) recommended that coaches and sport psychologists prioritize enhancing athletes' task-involvement instead of ego-involvement. They also emphasized considering motivational goals and self-confidence interactions when assessing emotional responses. Lastly, both groups converged on enjoyment as a core psychological need, aligning with the premise that rediscovering the pleasure of sport and maintaining a positive attitude enhances overall commitment (Fry and Gano-Overway, 2010).

Finally, addressing RQ 4, regarding the quantitative ratings of wellbeing and exploratory subgroup differences, the statistical data reinforced the qualitative narratives. Athletes' assessments revealed a low level of happiness within the team (*M* = 5.6 on a 10-point scale), which strongly correlated with team mental health, alongside a high team stress level (*M* = 7.3). Subgroup analyses yielded intriguing findings: staff members‘ evaluations showed a gender effect, with women reporting training less and experiencing higher stress levels compared to men (aligning with Murata et al., 2023). Interestingly, preliminary data suggest that individuals engaged in dual careers reported higher levels of happiness compared to those with a single career, contrasting with Conde et al. (2021), who found higher levels of negative emotional states among Spanish dual-career students during the COVID-19 pandemic.

The psychosocial and vocational layers of Wylleman's (2019) framework explain why individual symptoms, such as anxiety or low happiness, are deeply intertwined with the wider systemic dynamics, such as dual-career logistical strains and staff stress. However, the relationship between career engagement and wellbeing is a compelling area of inquiry that necessitates further exploration.

In conclusion, the primary contribution of this study was addressing a critical gap in the literature by providing a transparent, practical example of how to approach team-level needs assessment in applied sport psychology. By highlighting the disconnects between individual psychological distress (e.g., self-confidence, anxiety) and collective structural challenges (e.g., team cohesion, unshared goals, staff stress), this research underscored that sport psychology interventions cannot rely on a one-size-fits-all approach. Instead, they must be empirically guided by a structured intake protocol that captures the baseline realities of both the athletes and the performance staff.

## 5 Limitations and future perspectives

While this study offered valuable insights into team-level intake assessments, several limitations must be acknowledged. First, the interpretation of our findings, particularly the quantitative demographic comparisons regarding gender and dual-career status, was constrained by the small overall sample size and the limited number of staff participants. Consequently, these statistical analyses should be viewed as preliminary and interpreted with caution. Second, the study was characterized by the cultural specificity of the sample and setting, focusing exclusively on a single sport (i.e., elite women's rugby). This context-specific design inherently limited the generalizability of the findings to other athletic populations, competitive levels, or cultural environments.

Despite these limitations, the use of qualitative analysis remains increasingly popular in sport psychology and other social sciences, as it allows investigators to explore complex issues that are difficult to access through other methods and facilitates relationships and empathy between the sport psychologist, athletes, and staff members (Carrera-Fernández et al., 2014). However, qualitative research methods also present notable challenges, such as being time-intensive, susceptible to researcher bias, and lacking standardized criteria for evaluating findings (Smith and Eatough, 2006). To overcome these limitations, Marecek (2003) recommends embracing methodological pluralism and flexibility. Accordingly, this study adopted a mixed-methods approach by integrating quantitative measures alongside qualitative analysis to ensure a robust protocol.

Future research should address these methodological constraints by conducting larger-scale, multi-center studies across diverse sports, genders, and cultural contexts to enhance generalizability. Furthermore, replicating this protocol longitudinally within club settings, where routine on-field observation is possible, could yield additional valuable information on how baseline needs evolve over the course of a competitive season.

## 6 Practical implications

The lack of standardized procedures in the scientific literature for assessing and supporting elite rugby athletes represents a significant opportunity for further research and development in sport psychology, particularly in the context of women athletes' specific needs. Moreover, many of the physical, technical, tactical, and psychological programs used by professionals working in elite women's rugby are based on studies conducted with men athletes (Murata et al., 2023), perpetuating a well-documented 'sex and gender data gap' in sport and exercise science that overlooks the unique physiological and psychological realities of sportswomen (Cowley et al., 2021). A growing focus on women's sport is slowly being reflected in research. This study represented a step in that direction.

## 7 Conclusions

This study provided a description and the results of a participant-driven, whole-team intake assessment involving both athletes and multidisciplinary staff members in elite women's rugby. The assessment identified team cohesion and communication, individual self-confidence and emotion regulation, and mental-health and dual-career demands as relevant priorities. At a methodological level, the study demonstrated how qualitative accounts and structured self-report ratings can be combined to formulate context-specific psychological objectives rather than relying on generalized profiles. The findings may help sport psychologists, coaches, and sport organizations to distinguish individual, team, and organizational priorities and to establish appropriate performance-support and mental-health referral pathways. Because no intervention was evaluated and the subgroup analyses were exploratory, larger, multi-team, longitudinal studies are needed to test the transferability of these findings and evaluate subsequent interventions.

## Data Availability

The datasets presented in this article are not readily available because Data are not available due to ethical restrictions since they contain information that could compromise the privacy of research participants. Requests to access the datasets should be directed to alessia.pecchini@univr.it.
