# Supplementary material for: Listening first: mapping the inner game of an elite women's rugby national team and its staff
Source: Front Psychol. 2026 Sep 23;17:1927634. doi: 10.3389/fpsyg.2026.1927634 (PMC13642172; doi:10.3389/fpsyg.2026.1927634)
Supplement: Supplementary file 1 [file Supplementary_file_1.DOCX]

**Supplementary Material 1**

**Sport Interviewing Protocol (Adapted from Taylor, 2017)**

**ATHLETE’S VERSION**

*If you don’t mind, we ask your permission to record/video-record the interview.*

1. **Previous Experience with Sport Psychology**

- What, if any, has been your experience with sport psychology?
- What do you hope to get out of our work?

2. **Athletic and Life Goals**

- What are your personal athletic/life goals this season?

For example: physical (ie sleep, eating, illness, injury), athletic (physical conditioning, technique, tactics, quality and quantity of training), psychological (ie changes in cognition, mood, emotions, motivation, confidence, competition (routine pre and post competition, performance level)

What are the athletic/life goals for the team this season?

3. **Needs Assessment**

- Which aims do you expect psychological counselling will be focused on, for you?
- Which aims do you expect psychological counselling will be focused on, for the team?

For example, motivation, confidence, self-talk, intensity (i.e., arousal, energy, anxiety), focus, emotions, mental imagery, routines, perfectionism, fear of failure, need for control, communication, cohesion, roles respect, cooperation, goal setting.

4. **Physical Health**

- How would you rate your general physical health (1-10 scale)?
- How would you rate your general level of training (1-10 scale)?
- How would you rate your general level of sleep (1-10 scale)?
- How would you rate your eating behavior (1-10 scale)?

5. **Mental Health**

Personal

- How would you rate your general level of mental health (1-10 scale)?
- How would you rate your general happiness (1-10 scale)?
- How would you rate your general level of life stress (1-10 scale)?
- How would you rate your ability to cope with life stress (1-10 scale)?

Team

- How would you rate the general level of mental health (1-10 scale)?
- How would you rate the general happiness (1-10 scale)?
- How would you rate the general level of life stress (1-10 scale)?
- How would you rate the ability to cope with life stress (1-10 scale)?

6. **Family Background and Social Support**

Family

- How satisfied are you with the support you receive from your family within sport (1-10 scale)?
- How many and what quality of friendships do you have within sport?
- How many and what quality of friendships do you have outside sport?
- What are other sources of support you have?

National coaches

- How satisfied are you with the support you receive from your national coaches (1-10 scale)?
- How would you describe your relationship with your national coaches?
- How satisfied are you with the support you receive from your national assistant coaches (1-10 scale)?
- How satisfied are you with the support you receive from your national physical trainer (1-10 scale)?
- How satisfied are you with the support you receive from your national physiotherapist (1-10 scale)?
- How satisfied are you with the support you receive from your national doctors (1-10 scale)?
- How satisfied are you with the support you receive from your national team manager (1-10 scale)?

National teammates

- How satisfied are you with the support you receive from your national teammates (1-10 scale)?
- How would you describe your relationship with your national teammates?

**STAFF MEMBERS’S VERSION**

*We kindly ask your permission to audio/video-record the interview.*

1. **Previous Experience with Sport Psychology**

- What, if any, has been your experience with sport psychology? For example, have you ever attended a workshop, taken a course, or worked with a sport psychology consultant before?
- What do you hope to get out of our work?
- Do you have any concerns about our work?

2. **Professional Goals**

- What is your professional goal for this season?
- What are some of your process goals (that is, what you need to do to accomplish your outcome goals)?
- Using the SWOT analysis, what can you tell us about your goals?

3. **Needs Assessment**

- Which aims do you expect psychological counselling will be focused on, for the staff?
- Which aims do you expect psychological counselling will be focused on, for the players?

For example, motivation, confidence, self-talk, intensity (i.e., arousal, energy, anxiety), focus, emotions, routines, perfectionism, fear of failure, need for control, communication, cohesion, role cooperation, goal setting.

4. **Family Background and Social Support**

- Who gives you support in your sport (family, friends, others…)?
- How satisfied are you with the support you receive from your family, friends, and others (1-10 scale)?

5. **Physical Health**

- How would you rate your general physical health (1-10 scale)?
- How would you rate your general level of physical activity (1-10 scale)?
- How would you rate your general level of sleep (1-10 scale)?
- How would you rate your eating behavior (1-10 scale)?

6. **Mental Health**

- How would you rate your general level of mental health (1-10 scale)?
- How would you rate your general happiness (1-10 scale)?
- How would you rate your general level of life stress (1-10 scale)?
- How would you rate your ability to cope with life stress (1-10 scale)?
